# Targeting chemokine receptors in disease – a case study of CCR4

**DOI:** 10.1016/j.ejphar.2015.05.018

**Published:** 2015-09-15

**Authors:** Roberto Solari, James E. Pease

**Affiliations:** a Airway Disease Infection Section, MRC-Asthma UK Centre in Allergic Mechanisms of Asthma, National Heart and Lung Institute, Imperial College London, Norfolk Place, London W2 1PG, United Kingdom; b Leukocyte Biology Section, MRC-Asthma UK Centre in Allergic Mechanisms of Asthma, National Heart and Lung Institute, Imperial College London, South Kensington Campus, London SW7 2AZ, United Kingdom

**Keywords:** Chemokine, Receptor, Signaling, Antagonist, Inflammation, Asthma leukaemia

## Abstract

Since their early 1990s, the chemokine receptor family of G protein-coupled receptors (GPCRs) has been the source of much pharmacological endeavour. Best known for their key roles in recruiting leukocytes to sites of infection and inflammation, the receptors present themselves as plausible drug targets for therapeutic intervention. In this article, we will focus our attention upon CC Chemokine Receptor Four (CCR4) which has been implicated in diseases as diverse as allergic asthma and lymphoma. We will review the discovery of the receptors and their ligands, their perceived roles in disease and the successful targeting of CCR4 by both small molecule antagonists and monoclonal antibodies. We will also discuss future directions and strategies for drug discovery in this field.

## 1 Introduction

### 1.1 The chemokine superfamily

The chemokine superfamily of proteins serves to coordinate a variety of immune system functions that link both innate and adaptive immunity (Zlotnik and Yoshie, 2012) and is best known for its key role in the recruitment and retention of leukocyte populations in both homeostasis and immune responses to pathogens (Viola and Luster, 2008). The superfamily consists of over twenty GPCRs and around 40 chemokine ligands (Table 1) which act as receiver and signal respectively, to guide leukocytes between tissue compartments (Bachelerie et al., 2013). Movement of leukocytes is along a gradient of chemokine, a process known as chemotaxis, which is sensed by the cell surface chemokine receptors. Binding of the chemokine ligand to the extracellular face of the receptor activates multiple intracellular signaling pathways, with *Pertussis* toxin-sensitive G proteins of the G_αi_ subset and Phosphatidylinositol-3 kinases (PI3K) playing key roles.

### 1.2 Aspects of signaling

**Fig. 1:**
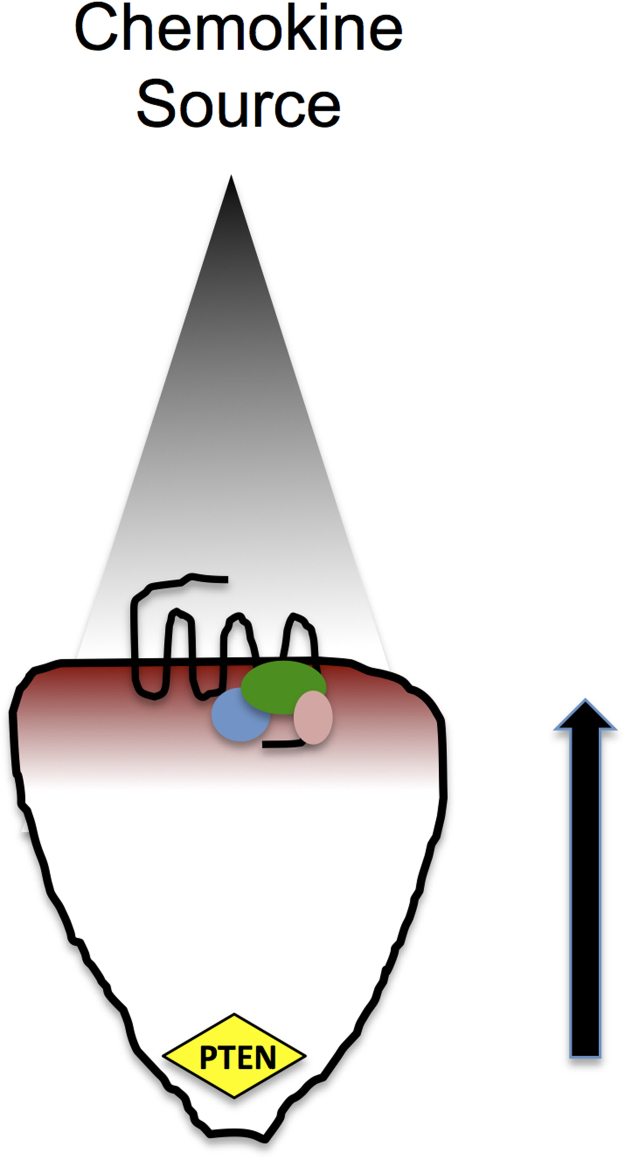
Cartoon showing a leukocyte migrating along a gradient of chemokine (gray) in the direction of the arrow. Activation of the 7TMRs at the leading edge of the cell activated PI3K to PIP_3_. The location of the phosphatase PTEN at the rear of the cell results in an internal gradient of PIP_3_ , which is concentrated in the vicinity of the receptor. This facilitates localized binding of Rho GTPases such as Cdc42 and Rac (blue, green pink ovals) which modulate the actin cytoskeleton and contribute to membrane protrusion in the direction of the chemokine source.

Activation of chemokine receptors leads to the recruitment of PI3K to the plasma membrane and the localized production of Phosphatidylinositol (3,4,5)-trisphosphate (PI (3,4,5) P_3_) from Phosphatidylinositol (4,5)-bisphosphate (PI (4,5) P_2_). The redistribution of the PI (3,4,5) P_3_ –specific phosphatase PTEN to the rear of the cell results in the generation of an intracellular gradient of PI (3,4,5) P_3_, which is highly enriched at the leading edge of the cell. This results in the localization of cytosolic proteins with pleckstrin homology (PH) domains which readily bind PI (3,4,5) P_3_ (Lemmon, 2007). Such proteins include Akt, GTPase activating proteins (GAPs) and guanine-nucleotide-exchange factors (GEFs). GEFs serve to activate small GTPases belonging to the Rho family by stimulating the exchange of GDP for GTP, thereby activating the protein. Activated Rho GTPases such as Cdc42 and Rac are well known for their contribution to membrane protrusion at the leading edge of the cell and the maintenance of cell polarity (Weiner et al., 2002) in conjunction with members of the Wiskott-Aldrich Syndrome Protein family, such as WASP (Symons et al., 1996). In contrast, GAPs enhance the intrinsic GTPase activity of the Rho family members, hydrolyzing GTP to GDP and turning off the protein function. Accordingly, actin polymerization at the leading edge of the cell, coupled with contraction at the rear, results in movement of the leukocyte in the direction of the stimulus, i.e. along the concentration gradient (Fig. 1).

Migration proceeds as long as the chemokine receptor maintains intracellular signaling and actin remodeling at the leading edge. The process is terminated by G protein receptor kinases (GRKs) which induce C-terminal phosphorylation of the chemokine receptor (Penela et al., 2014). This increases the affinity for the scaffold proteins of the arrestin family, which targets the receptor for clathrin-coated pit mediated endocytosis and degradation or recycling. Thus as the cell encounters increasing concentrations of chemokine, fewer and fewer receptors remain at the cell surface to drive the intracellular signals needed for chemotaxis and migration is inhibited. This is one potential explanation for the bell-shaped chemotaxis plots observed in dose–response experiments with increasing concentrations of chemokine. Once thought to function solely as a means of sterically hindering GPCR signaling and promoting receptor endocytosis (Lefkowitz, 1998), arrestin binding to GPCRs is now appreciated to induce additional signaling programs by acting as a scaffold for the recruitment of further signaling molecules such as MAP kinases (DeWire et al., 2007, Lefkowitz and Shenoy, 2005). The significance of this in terms of chemokine signaling was ably shown when mice deficient in arrestin-2 were shown to have impaired chemotaxis to CXCL12 (Fong et al., 2002). This has been translated in part to the human setting by studies of CXCR4 in patients suffereing from WHIM (Warts, Hypogammaglobulinemia, Infections, and Myelokathexis) syndrome where arrestin-2 dependent phosphorylation of ERK1/2 has been reported to account for the hyperresponsiveness of the receptor to its ligand CXCL12 (Lagane et al., 2008).

### 1.3 Biased agonism at chemokine receptors

Chemokines are typically promiscuous, binding to several receptors, with each receptor often having multiple ligands. This apparent redundancy was originally thought to provide a means by which robust responses to infectious agents could be generated *in vivo* (Mantovani, 1999, Zlotnik and Yoshie, 2012). In recent years, however, as different aspects of GPCR signaling have become appreciated, it is apparent that different ligands of the same GPCR can transduce signals via distinct cellular pathways leading to distinct signaling outputs. This is termed functional selectivity or biased agonism (Kenakin and Miller, 2010, Kenakin, 2012). The predominant pathway at which ligands diverge appears to be the arrestin-mediated signaling pathway. Several GPCRs exhibit biased agonism with respect to arrestin signaling, including the M3-muscarinic receptor (Poulin et al., 2010), histamine H4 receptor (Rosethorne and Charlton, 2011), vasopressin receptors (Rahmeh et al., 2012) and angiotensin II–type 1 receptors (Saulière et al., 2012). In the chemokine field, the CCR7 ligands CCL19 and CCL21 although equally active in assays of chemotaxis, have been shown to diverge at the level of receptor endocytosis (Bardi et al., 2001, Otero et al., 2006), arrestin-recruitment (DeWire et al., 2007, Kohout et al., 2004) and receptor desensitization (Penela et al., 2014, Zidar et al., 2009). We have recently uncovered aspects of biased signaling at the chemokine receptor CCR4, in both leukocytes and lung epithelial cells, which we believe to be of significance in the setting of allergic inflammation, more of which later (Ajram et al., 2014, Viney et al., 2014).

### 1.4 Targetting chemokines and their receptors

The inadvertent or over expression of chemokines has been implicated in just about every disease process with an inflammatory component, from diseases as seemingly diverse as asthma, atherosclerosis, multiple sclerosis and rheumatoid arthritis (Charo and Ransohoff, 2006, Viola and Luster, 2008). This has led to the notion that therapeutic intervention, in the form of chemokine receptors blockade may provide a novel therapeutic angle. The discovery that chemokine receptors are portals for the entry of HIV-1 into leukocytes (Alkhatib et al., 1996, Feng et al., 1996) has fueled the drug discovery process further, with inhibitors of the two major receptors, CCR5 (on macrophages) and CXCR4 (on T cells) highly prized. At the time of writing, two small molecule antagonists of CCR5 and CXCR4 have received approval by the relevant agencies. Miraviroc/Selsentri a CCR5 inhibitor from Pfizer has been licensed for the treatment of HIV-1 infection (MacArthur and Novak, 2008). Plerixafor, a CXCR4 antagonist originally developed for similar purposes, has been licensed for its ability to mobilize stem cells from the bone marrow, of use following administration of chemotherapeutics (Brave et al., 2010) and is also showing early promise as a treatment for patients with the immunosuppressive WHIM syndrome, resulting from dysregulation of CXCR4 function (McDermott et al., 2011).

In this article, we will focus upon the chemokine CCR4 and its ligands CCL17 and CCL22, which are postulated to play key roles in the pathogenesis of allergic asthma (Pease and Horuk, 2014), atopic dermatitis (Yamanaka and Mizutani, 2011) and a variety of cancers, including breast cancer (Li et al., 2012), gastric cancer (Yang et al., 2011) renal cell cancer (Liu et al., 2014) and lymphoma (Ishida and Ueda, 2011).

### 1.5 CCR4 – Discovery and initial characterization

The human coding sequence for CCR4 was first identified by the PCR amplification of a fragment from a cDNA library made from the basophilic cell line KU-812 and found to have around 50% homology to two other CC chemokine receptors identified at that time, CCR1 and CCR2 (Power et al., 1995). The original report assigned CCL3 as a functional ligand for CCR4, inducing Ca^2+^ influx in *Xenopus* oocytes although this may be an artifact of the system employed, since the authors subsequently showed that HEK-293 transfectants were unresponsive to CCL3 and its close relative CCL5 (Blanpain et al., 2001). Work from the group of Osamu Yoshie identified a transcript constitutively expressed in thymus and also by PBMCs following activation with phytohaemagglutinin, which they named Thymus and Activation-Regulated Chemokine (TARC) (Imai et al., 1996) and which they subsequently showed to be a high-affinity ligand for CCR4, inducing chemotaxis and Ca^2+^ influx in CCR4 transfectants (Imai et al., 1997). Northern blot analysis in the same manuscript showed CCR4 mRNA to be expressed by human CD4^+^ T cells and a handful of T-cell lines including Hut-78 and Jurkat. Expression of CCR4 has subsequently been demonstrated on several T-cell subsets including Th2 and T regulatory (Treg) cells (discussed later) and more recently on Th17 (Acosta-Rodriguez et al., 2007, Lim et al., 2008) and Th22 cells (Trifari et al., 2009), where it is co-expressed with other chemokine receptors , notably CCR6.

Shortly after the discovery of TARC, another CC chemokine was cloned independently by two groups. The group of Patrick Gray at ICOS Corporation named their discovery “monocyte derived chemokine” (MDC) since it was expressed by macrophages and monocyte-derived dendritic cells (Godiska et al., 1997). Andrew and colleagues at Amgen simultaneously cloned an identical CC chemokine by EST sequencing of a cDNA library prepared from activated macrophages which they named STCP-1 (Stimulated T-cell chemotactic protein) since it recruited T-cells in chemotaxis assays (Chang et al., 1997). Both groups subsequently showed that the chemokines bound to the same chemokine receptor, namely CCR4 (Andrew et al., 1998, Imai et al., 1998). TARC and MDC/STCP-1 are now known as CCL17 and CCL22 respectively (Zlotnik and Yoshie, 2000). Both CCL17 and CCL22 bind CCR4 with low nanomolar affinity and have similar potencies in chemotaxis assays, although CCL22 is the slightly more efficacious ligand of the two. The genes for CCL17 and CCL22 reside in close proximity on human chromosome 16q13 suggesting that they arose by gene duplication, although the mature protein sequences are less than 40% identical (Imai et al., 1998). Both CCL22 and CCL17 are expressed in the thymus leading to the notion that one role of the receptor may be to regulate the intrathymic movement of CCR4^+^CD4^+^CD8^+^ thymocytes during the process of T lymphocyte education and differentiation (Annunziato et al., 2000, Chantry et al., 1999).

### 1.6 CCR4 and its ligands in disease

#### 1.6.1 Asthma

A considerable body of evidence points to a role for CCR4 and its ligands in allergic diseases, notably asthma. Polarization of human T-cells *in vitro* to Th2 subsets by culture with cytokine and antibody cocktails (IL4, anti-IFNγ and anti-IL-10), has been well documented to generate IL-4 producing Th2 cells, which express CCR4 at both protein and message level (Bonecchi et al., 1998, Sallusto et al., 1998). This facilitates their recruitment by dendritic cells which produce CCL17 and CCL22 during maturation (Tang and Cyster, 1999). Upregulation of CCR4 on T cells is mirrored *in vivo*, with CCR4 expression a key feature of IL-4 producing T cells recovered from the bronchoalveolar lavage fluid of asthmatic and healthy subjects CCR4 (Morgan et al., 2005, Panina-Bordignon et al., 2001). The CCR4 ligands CCL22 and CCL17 are also upregulated in the lung following allergen challenge (Bochner et al., 2003, Pilette et al., 2004). More recently, a study by Vijayanand and colleagues demonstrated increased CCR4 expression on T cells isolated from patients with asthma. They also notably demonstrated that CCL17 but not CCL22 was significantly upregulated following challenge of *ex vivo* airway biopsies with house dust mite extract (Vijayanand et al., 2010).

A role for CCR4 expression on bronchial epithelial cells has been discovered (Bonner et al., 2013). Interestingly, previous studies reported that bronchial epithelial cells in culture can also produce CCL17 (Sekiya et al., 2000), highlighting the potential for a positive feedback signaling loop. We have recently shown that CCR4 is expressed by both primary bronchial epithelial cells and lines such as BEAS2B, and can bind and internalize CCR4 in response to ligand. Notably, we observed that CCL17 was an extremely efficacious inducer of α-CGRP synthesis and release (Bonner et al., 2013). This, we hypothesize, may play a pathological role in asthma, since α-CGRP production is markedly increased in the airways of asthmatic patients challenged with allergen-derived T-cell peptides (Kay et al., 2007). α-CGRP is known to act synergistically with other mediators of inflammation, including histamine, to produce marked and prolonged edema, thus contributing to pathology (Brain and Williams, 1985). Curiously, in contrast to CCL17, CCL22 is a feeble inducer of α-CGRP release, with an approximately 10,000-fold reduction in activity compared to CCL17, despite both ligands binding CCR4 with similar affinity (Imai et al., 1998, Imai et al., 1997). To our knowledge, this is the first published description of a definitive physiological outcome by an endogenous biased agonist of a chemokine receptor.

Data obtained in humans has been corroborated to a certain extent by rodent models of allergic airways disease. Mikhak et al showed that antigen-specific Th2 cells adoptively transferred from CCR4-deficient mice fail to traffic in significant numbers to the allergic lung (Mikhak et al., 2009). Similarly, antibody neutralization of either CC17 or CCL22 proved to be effective in reducing leukocyte recruitment to the lung and associated parameters of inflammation, following allergen challenge (Kawasaki et al., 2001, Lloyd et al., 2000). In contrast to these studies, ovalbumin challenged CCR4 null mice were not protected against airways inflammation compared with littermates (Chvatchko et al., 2000), nor were ovalbumin challenged guinea pigs protected from lung inflammation when CCR4 was neutralized by an antibody (Conroy et al., 2003). This suggests that the underlying biology of CCR4 may be subtly different in rodents and man. A recent study circumvented this by using a human PBMC-reconstituted SCID mouse model and found that CCR4 blockade via a specific antibody ablated many of the features of inflammation, including airway eosinophilia, goblet cell hyperplasia, IgE synthesis and bronchial hyper-reactivity, thus reinforcing the idea that CCR4 is a viable target in the treatment of asthma (Perros et al., 2009).

#### 1.6.2 CCR4 and its ligands in allergic dermatitis

The discovery by Campbell and colleagues that skin-homing cutaneous lymphocyte antigen (CLA)^+^ T cells express high levels of CCR4 expression (Campbell et al., 1999), implicated the receptor in the pathology of atopic dermatitis. This was subsequently supported by a study in which the levels of both CCR4 and CLA were shown to be increased on the surface of peripheral blood CD4^+^ T cells from severe atopic dermatitis subjects compared with control subjects. CCR4 expression levels were also shown to decrease as disease symptoms improved (Wakugawa et al., 2001). In mouse models of cutaneous delayed type hypersensitivity, CCR4 has been shown to support the homing of T cells to skin (Reiss et al., 2001). Several studies have reported elevated serum levels of CCL17 in human atopic dermatitis subjects (Shimada et al., 2004) with CCL17 thought to be produced by keratinocytes (Vestergaard et al., 2000). Serum levels of the chemokine show close correlation with disease severity (Kakinuma et al., 2001) . Indeed, out of a panel of adult biomarkers, CCL17 was found by Kou et al to have the highest odds ratio for the likelihood of having atopic dermatitis (Kou et al., 2012). This is consistent with *in vitro* studies showing that corneal and dermal fibroblasts stimulated with the Th2-associated cytokines IL-4 and IL-13 are important sources of CCL17 (Fukuda et al., 2003). Human platelets have also been shown to express CCR4 and to undergo aggregation following stimulation with CCL17 and CCL22 (Abi-Younes et al., 2001, Clemetson et al., 2000, Kowalska et al., 2000). This was postulated by Abi-Younes and colleagues to explain the increased serum levels of CXCL4 in a murine model of atopic dermatitis (Watanabe et al., 1999), since CXCL4 is a marker of platelet degranulation. Moreover, since platelets have been shown to contain CCL17 there is potential for positive feedback in this process (Fujisawa et al., 2002).

#### 1.6.3 CCR4 and its ligands in T-cell neoplasms

Working with the hypothesis that the chemokine expression pattern of T-cell neoplasm may give insight into their cellular origins, the group of Osamo Yoshie were first to show that CCR4 was expressed at consistently high levels on the surface of a wide range of human T-cell lines (Yoshie et al., 2002), including Hut87 and Jurkat lines, which they had previously examined by Northern blot analysis (Imai et al., 1997). Using a panel of 24 adult adult T cell lymphoma patients, the vast majority of PBMCs from these subjects (22/24) were shown to express CCR4 by PCR and to respond to CCL17 and CCL22 in chemotaxis assays (Yoshie et al., 2002). Subsequent studies by others confirmed the expression of CCR4 in archived adult T cell lymphoma tissues (Ishida et al., 2003) and also showed CCR4 to be abundantly expressed by other neoplasms including some peripheral T cell lymphoma and NK cell lymphomas (Ishida et al., 2004) and cutaneous T cell lymphomas (Campbell et al., 2010, Ferenczi et al., 2002). Subsequent studies addressing the molecular mechanisms for CCR4 upregulation, found the transcription factor *FRA-2* to be significantly upregulated in adult T cell lymphoma and to drive enhanced CCR4 expression and proliferation of both adult T cell lymphomas and cutaneous T cell lymphoma s (Nakayama et al., 2007, Nakayama et al., 2012). A recent study has described mutations in the C-terminus of CCR4 that truncate the receptor and lead to a gain of function, with respect to enhanced cellular signaling, chemotaxis and proliferation (Nakagawa et al., 2014). These are remarkably similar to truncating mutations in CXCR4 that have been described as exacerbating signaling in WHIM syndrome patients (Hernandez et al., 2003).

## 2 Blockade of CCR4 in the treatment of disease

### 2.1 Anti-CCR4 biologicals

Monoclonal antibodies targeting CCR4 have been described by several groups. Our own group, in collaboration with scientists at LeukoSite/Millennium Pharmaceuticals characterized a panel of CCR4-specific antibodies which were generated by immunization of mice with transfectants expressing the receptor (Andrew et al., 2001). The most promising of these was a molecule known as 10E4 which recognizes an N-terminal epitope of CCR4 (Jopling et al., 2002) and which we subsequently used to neutralize CCR4 in a guinea pig model of allergic airways disease (Conroy et al., 2003). Scientists at Kyowa Hakko Kogyo generated a murine monoclonal antibody named KM-2160 by immunizing mice with a peptide corresponding to amino acid residues 2–29 of the human CCR4 N-terminus (Ishida et al., 2003). Using this mAb, they showed that increased levels of CCR4 staining on primary adult T cell lymphoma cells correlated with decreased survival of the patient. This finding spurred on the authors to assess the efficacy of this antibody in mediating antibody-dependent cellular cytotoxicity, turning CCR4 into a target by which adult T cell lymphomas could be sought out and destroyed by host NK cells. The antibody underwent subsequent modifications, namely cDNAs encoding the heavy- and light chain variable region of the KM-2160 hybridoma were cloned into an IgG1 antibody expression vector and the construct expressed in the rat myeloma cell line YB2/0. From the supernatant they were able to purify a chimeric anti-CCR4 antibody which they named KM-2760 (Niwa et al., 2004). This antibody has low levels of fucosylation in the Fc region (7%), which they showed corresponded to greater activity in antibody-dependent cellular cytotoxicity assays. KM2760 then underwent full humanization to generate the mAb KW-0761 (also known as mogamulizumab) which demonstrated potent antitumor activity against primary adult T cell lymphomas both *in vitro* and *ex vivo* (Ishii et al., 2010).

Mogalizumab subsequently entered clinical trials for the treatment of both adult T cell lymphoma and peripheral T cell lymphoma and was found to be well tolerated, have a half life of around 18 days and meet preliminary objective responses (Yamamoto et al., 2010). Subsequent phase II studies of relapsed adult T cell lymphoma patients (Ishida et al., 2012) and relapsed peripheral T cell lymphomas and cutaneous T cell lymphomas (Ogura et al., 2014) found mogalizumab to again show efficacy, with significant numbers of objective responses seen in all patient groups. In 2012, mogalizumab was granted approval for the treatment of relapsed or refractory adult T cell lymphoma in Japan.

### 2.2 Small molecule antagonists of CCR4

Given the importance of CCR4 and its ligands in allergic inflammatory diseases there has been a significant effort over many years to discover small molecule CCR4 antagonists. However, despite all these endeavors, so far only one molecule has made it to human clinical trials and that too appears to have been terminated (Cahn et al., 2013, Solari et al., 2014). Chemokine receptors are GPCRs, which are historically the most successful target class for drug discovery, so this lack of success has been surprising and has been attributed to many factors (Solari et al., 2014).

**Fig. 2:**
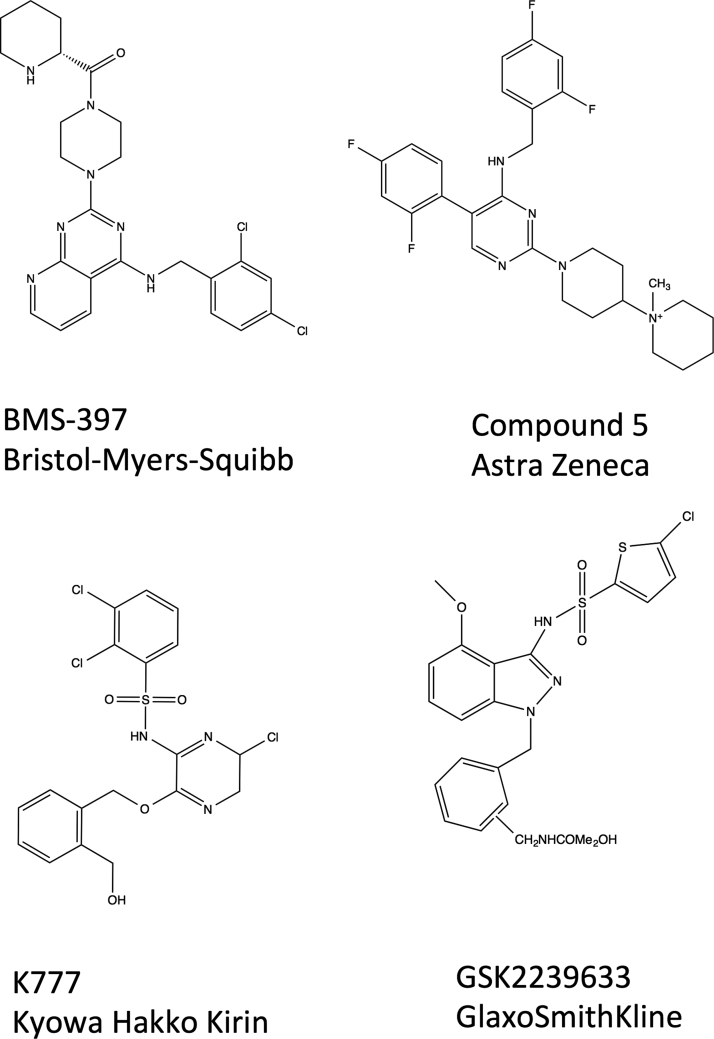
The chemical structures of a handful of small molecule CCR4 antagonists described in the main text.

The patent literature for CCR4 antagonists began to emerge around 2002 and the first comprehensive review of the field in 2006 revealed that these could be divided into four main groups; aryl sulphonamides, substituted amino heterocycles, thiazolidinones and lactams (Purandare and Somerville, 2006). Since then there have been many reports of CCR4 antagonists that can grouped into two main chemical categories. The first is a collection of lipophilic heteroarenes from Bristol Myers Squibb, Astellas and Daiichi Sankyo and the second is a range of aryl sulphonamides from Astra Zeneca, Ono and GlaxoSmithKline (Andrews et al., 2007, Banfield et al., 2010, Burdi et al., 2007, Cahn et al., 2013, Kuhn et al., 2007, Nakagami et al., 2010a, Nakagami et al., 2010b, Nakagami et al., 2009, Procopiou et al., 2013, Procopiou et al., 2012, Purandare et al., 2007, Solari et al., 2014, Yokoyama et al., 2009, Yokoyama et al., 2008, Zhao et al., 2009). Some pertinent structures from these studies are shown in Fig. 2. A number of these compounds looked very promising and showed efficacy in animal models of allergic inflammation however only one, an indazole arylsulfonamide, GSK 2239633 (Slack et al., 2013) appears to have progressed to clinical trials (Cahn et al., 2013).

**Fig. 3:**
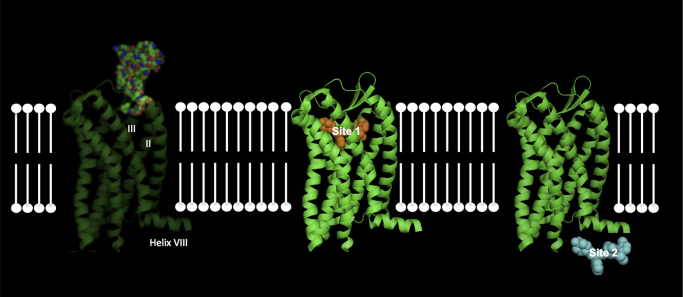
A diagram representing various modes of allosteric modulation at CCR4. The left panels shows transmembrane helices II and III of CCR4 being activated by chemokine (space filled model). The centre panel shows a "Site 1" antagonist (yellow) bound to an intrahelical binding site. The right panel shows a lipophillic "Site 2" antagonist (cyan) interacting with C-terminal helix VIII of CCR4.

Clues began to emerge about the complex biology of CCR4 that might explain why drug discovery has been so challenging. The first came from studies by Astra Zeneca on a series of pyrazinyl-sulfonamides that were allosteric antagonists of CCR4 and that appeared to bind to an intracellular site on the receptor, the so-called “Site 2” (Andrews et al., 2007). Furthermore, it appeared that this intracellular allosteric binding site was different to the site bound by the compound BMS-397 (“Site 1”) and both of these antagonist sites were distinct from the binding site for the natural ligands (Ajram et al., 2014). Unpublished mutagenesis work from our groups suggests that “Site 1” resides in a well characterized hydrophobic pocket comprised of resides in transmembrane helix III, whilst “Site 2” is centered around the cytoplasmic Helix VIII thought to run parallel to the lipid bilayer (Fig. 3).

In addition to this complex pharmacology it appears the receptor displays complex biological responses to its natural ligands. Like most GPCRs, CCR4 is internalised following agonist binding as part of the desensitisation process. However, it appeared that the two natural CCR4 ligands, CCL17 and CCL22, induced different rates of receptor internalisation (Imai et al., 1998, Mariani et al., 2004). Moreover, this difference in receptor trafficking was reflected by small molecule antagonists. Arylsulphonamides that bind to the intracellular allosteric site were unable to induce receptor internalisation whereas lipophilic amine antagonists binding to the extracellular site were (Ajram et al., 2014). The possibility that receptor down regulation by antagonists may contribute to the inhibition of a biological response was highlighted by studies of another CCR4 antagonist, K777 (Sato et al., 2013). Thus this chemokine receptor, and perhaps others, shows complex regulation of cell surface expression and trafficking that may in part reflect the need for accurate control of chemotaxis signals. In addition to differences in receptor trafficking, the two natural ligands also showed differences in receptor coupling with CCL22 coupling to arrestin signaling, whereas CCL17 does not (Ajram et al., 2014).

## 3 Conclusions

Clearly CCR4 is amenable to the discovery of drug-like antagonists, however the failure of these to translate small molecules into drugs raises the question that perhaps we still do not appreciate the subtle and complex biological controls that regulate the function of this receptor. For example, what are the relative contributions of arrestins to CCR4-mediated signalling? Which members of the GRK family govern CCR4 desentization and trafficking? An obvious potential caveat of total CCR4 blockade as an asthma treatment is the potential for the impairment of regulatory T cell recruitment, since T-regulatory cells (Tregs) have been shown to express CCR4 and to migrate *in vitro* in response to both CCL17 and CCL22 (Iellem et al., 2001). Blockade of CCR4 function on these cells might therefore be envisaged to worsen rather than dampen allergic inflammation since Tregs have the capacity to suppress Th2-mediated inflammation *in vivo* (Saito et al., 2008). Indeed, mogalizumab treatment has been associated with several cases of severe skin inflammation, notably Steven–Johnson syndrome, which in one case proved to be fatal (Ishida et al., 2013). Examination of one Steven–Johnson syndrome patient revealed a significant reduction in staining for the Treg marker FOXP3, in both PBMCs and skin lesions, incriminating Treg depletion in the pathogenesis. The efficacy of mogamulizumab in the treatment of CCR4-negative solid cancers, where Treg depletion is desirable, is currently being assessed. A recent report outlining the treatment of four elderly patients with mogalizumab has also suggested that an additional side-effect may be the risk of opportunistic infection with cytomegalovirus (Ohyama et al., 2014).

However, it may be possible to employ small molecules to block the activity of one CCR4 agonist whilst sparing another, since they are biased agonists with respect to arrestin coupling (Ajram et al., 2014) and also with respect to αCGRP induction in bronchial epithelial cells (Bonner et al., 2013). In a proof of principle approach, we have recently shown that CCL22 signaling can be spared whilst ablating CCL17 signaling by the use of a CCR4-specific mAb 10E4 which binds the receptor N-terminus (Viney et al., 2014). This mAb presumably preferentially inhibits a CCR4 conformation required for CCL17 signaling but dispensable for CCL22 signaling. This may be important in dialing out the off-target effects of inhibiting Treg recruitment, since their recruitment by activated dendritic cells appears to be mediated principally via CCL22 (Iellem et al., 2001). Analysis of the structure–activity relationship of existing compounds with attention to the signaling pathways blocked by the compounds could be a fruitful approach to fine-tune these compounds into molecules which block CCL17 but spare CCL22 signaling.

## Figures and Tables

**Table 1:** **Human chemokine receptors and their cellular expression.** Table showing the accepted cellular/tissue distribution of chemokine receptors and their ligands. Abbreviations: B, B-lymphocyte; Bro, Bronchial epithelial cells; Bs, basophil; DC, dendritic cell; Eo, eosinophil; Ker, keratinocytes; Mc, mast cell; Mo, monocyte; MSC, Mesenchymal Stem cell, NK, natural killer cell; No, neutrophil; NT, neuronal tissue; LEC, lymphatic endothelial cell; P, platelets; RBC, red blood cell; SLO, secondary lymphoid organ; Syn, Syncytiotrophoblast; T, T-lymphocytes; VEC, vascular endothelial cell (adapted from Pease, 2011).

**Chemokine receptor**	**Chemokine ligands**	**Cellular expression**	**Chemokine receptor**	**Chemokine ligands**	**Cellular expression**
CCR1	CCL3, CCL4, CCL5, CCL7, CCL8, CCL13, CCL14, CCL15, CCL16, CCL23	Mo, DC, Eo, Bs, T, PMN, NK	CXCR1	CXCL5, CXCL6, CXCL8	No, Mo
CCR2	CCL2, CCL5, CCL7, CCL8, CCL13, CCL16	Mo, DC, T, Bs	CXCR2	CXCL1, CXCL2, CXCL3, CXCL5, CXCL6, CXCL7, CXCL8	No, Mo
CCR3	CCL4, CCL5, CCL7, CCL11, CCL13, CCL15, CCL24, CCL26, CCL28	Eo, T, Bs, Mc	CXCR3	CXCL4, CXCL4L1, CXCL9, CXCL10, CXCL11,	T, B
CCR4	CCL17, CCL22	T, MC, Bro, NK, P	CXCR4	CXCL12	T, B, DC, Mo
CCR5	CCL3, CCL4, CCL5, CCL7, CCL14, CCL15	Mo, DC, T	CXCR5	CXCL13	T, B
CCR6	CCL20	DC, T	CXCR6	CXCL16	T
CCR7	CCL19, CCL21	DC, T, B, NK			
CCR8	CCL1, CCL18	Mo, T, NK	XCR1	XCL1, XCL2	T, NK
CCR9	CCL25	T			
CCR10	CCL27, CCL28	T	CX3CR1	CX3CL1	T, NK, DC, Mo
					
ACKR1 (DARC)	CCL2, CCL5, CCL7, CCL11, CCL13, CCL14, CCL17,CXCL5, CXCL6,CXCL11	RBC, VEC	ACKR2 (D6)	CCL1,CCL5, CCL7, CCL11, CCL13, CCL14, CCL17, CCL22	LEC, Syn
ACKR3 (CXCR7)	CXCL11, CXCL12	B, MSC, NT	ACKR4 (CCRL1)	CCL19, CCL21, CCL25	Ker, SLO
